# Sperm DNA Fragmentation in Normozoospermic Men Is Associated with Blastocyst Formation and Quality in Conventional In Vitro Fertilization

**DOI:** 10.3390/jcm14248892

**Published:** 2025-12-16

**Authors:** Yusaku Mori, Linji Chen, Shogo Nishii, Miwa Sakamoto, Makoto Ohara, Akihiko Sekizawa, Sho-Ichi Yamagishi

**Affiliations:** 1 Department of Diabetes, Metabolism, and Endocrinology, Showa Medical University Graduate School of Medicine, Tokyo 142-8555, Japan; 2 Department of Obstetrics and Gynecology, Showa Meical University Graduate School of Medicine, Tokyo 142-8555, Japan; 3 Department of Obstetrics and Gynecology, Beijing Friendship Hospital, Capital Medical University, Beijing 100052, China

**Keywords:** assisted reproductive therapy, blastocyst, in vitro fertilization, male infertility, sperm DNA fragmentation

## Abstract

**Background**: Assisted reproductive therapy (ART) has been utilized as an effective therapeutic strategy for addressing infertility worldwide, and one of the key determinants of ART success is the acquisition of high-quality embryos through in vitro fertilization (IVF). We investigated which male factors were associated with embryo formation and quality in conventional IVF (cIVF). **Methods**: This study was an exploratory subanalysis of a trial conducted to examine the associations of clinical and lifestyle factors with sperm abnormalities in 41 men from infertile couples without identifiable male factors. From the original cohort, 21 men whose partners underwent cIVF were included for blastocyte outcome assessment. Semen samples were evaluated for standard sperm parameters and DNA fragmentation index (DFI). Blood biochemical parameters and lifestyle habits were also evaluated. Blastocysts were assessed 5 days after cIVF, and implantation success was determined 10 days after embryo transfer. **Results**: All participants showed normozoospermia, with mild sperm DFI in 76%. Blastocysts were formed in 32% of the oocytes following cIVF, with good blastocyst development and quality observed in 71% and 39%, respectively. The implantation success and live birth rates after embryo transfer were 53% and 43%, respectively. Regression analysis identified sperm DFI as the only variable inversely associated with all blastocyst outcomes. In contrast, no parameters were associated with implantation success or live birth rates. **Conclusions**: The present subanalysis suggests the hypothesis that sperm DNA fragmentation may be negatively associated with high-quality embryo formation in cIVF, even among normozoospermic men with non-severe levels of sperm DFI.

## 1. Introduction

The declining birth rate has become a serious social issue in many developed countries, particularly those with a birth rate below 2.0 [1]. Epidemiological studies have indicated that the increasing rate of infertility may be one contributing factor to this trend [2,3,4]. Assisted reproductive technologies (ART) are widely used to support individuals who are unable to conceive naturally, and the number of births resulting from ART has been steadily increasing worldwide [2,3,4], thus highlighting the growing importance of ART for infertile couples.

In ART, oocytes and sperm are collected and fertilized outside the body through in vitro fertilization (IVF), and the resulting fertilized oocytes are further cultured to develop embryos, which are subsequently transferred to the uterus [5]. A cohort study reported that overall pregnancy success rate with ATR is approximately 28% in women aged 25 to 35 years, whereas success rate increases up to approximately 45–50% when embryo transfer is performed [4]. Furthermore, embryo development and quality are also closely associated with pregnancy outcomes following embryo transfer [6]. Therefore, the acquisition of well-developed embryos through IVF is one of the critical determinants of ART success.

While female factors affecting oocyte quality are well established, male factors influencing sperm may also be critical for fertilization and subsequent embryo development. Indeed, male factors are estimated to account for up to approximately 30–50% of infertility cases among couples [7,8]. However, the underlying causes of male infertility remain unidentified in most cases [7,8]. Furthermore, the specific male factors associated with successful fertilization and high-quality embryo development in IVF are not fully understood.

Recently, we have found in a prospective observational study that blood and semen oxidative stress markers are associated with reduced sperm count and motility in men from infertile couples without identifiable causes of male infertility [9]. In the present study, we conducted an exploratory subanalysis of this original cohort to investigate which male factors were associated with embryo formation and quality in conventional IVF (cIVF).

## 2. Materials and Methods

### 2.1. Ethics Statement

This study was a subanalysis of a trial conducted to examine the associations of clinical and lifestyle factors with sperm parameters [9]. The study design was approved by the Ethics Committee of Showa University (Approval No: 2023-047-B, approval date: 18 July 2023), and the outcomes of the present study were prespecified as secondary outcomes in the protocol. All procedures were performed in accordance with the ethical standards of Showa University policy on human experimentation and the Helsinki Declaration of 1964 and its later versions. Informed consent was obtained from all participants.

### 2.2. Study Participants

Our previous study enrolled 41 men from infertile couples without obvious male factor infertility who visited the Reproduction section of the Obstetrics and Gynecology Department at Showa University Hospital (Tokyo, Japan) from July 2023 to April 2024; the exclusion criteria are described elsewhere [9]. Of the 41 participants, 21 men whose partners underwent cIVF were included in the present analysis, as illustrated in the flow chart provided in Supplemental Figure S1. All participants were followed until October 2025.

### 2.3. Study Design

Analyses that were prespecified in the original study protocol were (1) blastocyst formation, blastocyst development, and blastocyst quality grade after cIVF; (2) implantation success and live birth rates after embryo transfer; and (3) their associations with male and female parameters. In this subanalysis, a data-driven evaluation of the association between sperm DNA fragmentation index (DFI) and male parameters was performed. However, this subanalysis was an exploratory, hypothesis-generating analysis, rather than a confirmatory one, due to the small sample size. After enrollment, participants underwent routine clinical and physical examinations and skin advanced glycation end product (AGE) measurement, completed a lifestyle habits questionnaire [10], and provided semen and blood samples under non-fasting conditions. Skin AGE levels were non-invasively measured as skin autofluorescence (SAF) with AGE-Reader™ Mu (Diagnoptics Technologies B.V., Groningen, The Netherlands) [10].

### 2.4. Lifestyle Questionnaire

Lifestyle habits were evaluated using the following 12 multiple-choice questions as described in a previous report [10]: (1) exercise frequency, (2) smoking duration, (3) alcohol consumption frequency, (4) sleep duration, (5) perceived mental stress, (6) vegetable intake, (7) breakfast frequency, (8) tendency to overeat, (9) greasy food consumption, (10) processed food consumption, (11) sugary food consumption, and (12) starting meals with vegetables. Each item was scored on a scale from 1 (worst) to 5 (best). Exercise-, smoking-, and alcohol-related habits were assessed using items (1), (2), and (3), respectively. Mental stress-related lifestyle habits were evaluated using the average score of items (4) and (5). Diet-related lifestyle habits were assessed by averaging the scores of items (6) through (12).

### 2.5. Blood Sample Measurements

Serum levels of oxidative stress, glucose, lipids, zinc (Zn), free testosterone, and anti-Müllerian hormone (AMH) were measured using the following methods, respectively: the d-ROMs test (Wismerll Company Limited, Bunkyo, Tokyo, Japan), an enzyme electrode method, colorimetric assay, atomic absorption spectrophotometry, radioimmunoassay, and an enzyme-linked immunosorbent assay [11]. Normal values are described previously [9].

### 2.6. Sperm Sample Measurement

Fresh semen samples were collected by masturbation after 2–7 days of sexual abstinence and immediately used for the assessment of sperm parameters, as previously described [9,12]. Semen oxidative stress levels were immediately evaluated by measuring the oxidation–reduction potential (ORP) of semen samples with the MiOXSYS™ analyzer (Aytu BioScience, Englewood, CO, USA) [13,14] within the same facility. A drop of fresh semen was placed on a glass slide to prepare sperm smears, and sperm DFI was measured in the stored smears using the terminal deoxynucleotidyl transferase dUTP nick end labeling (TUNEL) method. DFI was expressed as the percentage of TUNEL-positive sperm to the total sperm count [9]. Sperm DFI was classified as mild (<15%), moderate (15–30%), and severe (>30%) [15,16]. Normal reference values for sperm parameters were defined as follows based on WHO criteria and other previous reports [12,13,14]: semen volume of >1.4 mL; sperm concentration of >16 × 10^6^/mL; sperm number of >39 × 10^6^/ejaculate; total motility of ≥42%; progressive motility of ≥30%; and ORP of <1.34 mV/10^6^ sperm/mL.

### 2.7. Stimulation Protocols in cIVF

Controlled ovarian stimulation was performed using either a gonadotropin-releasing hormone (GnRH) antagonist or a progestin-based protocol. Recombinant follicle-stimulating hormone (FSH) was initiated on menstrual cycle days 2–4, with doses adjusted individually. In the GnRH antagonist protocol, a GnRH antagonist was added once the leading follicle exceeded 14 mm. In the progestin-based protocol, dydrogesterone was administered from the start of FSH stimulation. Final oocyte maturation was induced when at least two follicles reached ≥18 mm using a buserelin nasal spray combined with recombinant human chorionic gonadotropin (hCG), followed by standard oocyte retrieval.

### 2.8. cIVF Procedure

IVF was performed approximately 3 h after oocyte retrieval. On the day of IVF, fresh semen samples were collected separately from those used for sperm parameter assessment and were prepared using standard density gradient centrifugation. Motile sperm were resuspended in fertilization medium. Cumulus–oocyte complexes were cultured with sperm (100,000–150,000/mL) in HTF medium supplemented with serum substitute (Irvine Scientific, Irvine, CA, USA) at 37 °C under 5% O_2_, 6% CO_2_, and balance N_2_. Fertilization was assessed 16–18 h later by the presence of two pronuclei.

### 2.9. Blastocyst Assessment

Fertilized oocytes were further cultured to the blastocyst stage for cryopreservation and evaluated on day 5 using the Gardner and Schoolcraft grading system [17]. Blastocyst development stage was defined as: 0, pre-blastocyst; (1) blastocoel cavity occupying <50%; (2) blastocoel cavity occupying approximately 50% of embryo; (3) blastocoel cavity occupying >50%; (4) blastocoel cavity fully expanded with thinning zona pellucida; (5) partially hatching blastocyst; (6) completely hatched blastocyst. Blastocyst formation rate was calculated as the percentage of embryos reaching stage ≥1 among total cultured oocytes. The developmental stage was averaged across all cultured oocytes as an individual value. Blastocysts at stage ≥3 were further assessed for inner cell mass (ICM) and trophectoderm (TE) quality. ICM and TE were graded as follows: ICM: A = tightly packed distinct cells, B = loosely grouped cells, C = few/disorganized cells; TE: A = cohesive epithelium, B = fewer/looser cells, C = sparse/disorganized cells. Each blastocyst was classified as good (ICM and TE: AA, AB, BA), moderate (BB), low (BC, CB), or very low (CC) grade [6] and scored 3, 2, 1, or 0, respectively. The average score was calculated as an individual value. During cIVF and subsequent embryo transfer, male parameters were blinded except for age and routine sperm parameters such as semen volume, sperm concentration, total sperm count, and sperm total and progressive motility.

### 2.10. Embryo Transfer Procedure

Embryo transfer was performed under transvaginal ultrasound guidance using a soft embryo transfer catheter. All transfers were conducted in hormone replacement cycles. Endometrial preparation was initiated on day 2 of the menstrual cycle with estradiol and continued until endometrial thickness reached ≥8 mm on ultrasound. Once the target endometrial thickness was achieved, oral dydrogesterone (30 mg/day) and intravaginal progesterone (300 mg/day) were administered for 5 consecutive days before embryo transfer. A single embryo was selected for transfer based on morphological quality and developmental stage, typically on day 5. The selected embryo was loaded into the catheter in a minimal volume of culture medium and gently deposited approximately 1–2 cm from the uterine fundus. Implantation success was assessed 7 days after transfer by detecting serum hCG. The implantation success rate after transfer was calculated as follows: implantation success events/embryo transfer attempts × 100. Data on miscarriage and live birth were obtained from medical records. The miscarriage rate was calculated as the number of miscarriage events divided by the number of implantation successes × 100, and the live birth rate was calculated as the number of live births divided by the number of embryo transfer attempts × 100.

### 2.11. Statistical Analyses

Statistical analyses were performed with JMP Pro statistical software version 17.0.0 (SAS Institution Inc., Cary, NC, USA), except for Cronbach’s α, which was calculated with R software version R 4.4.2 (R Core Team; R Foundation for Statistical Computing, Vienna, Austria). The normality of data distribution was tested with the Shapiro–Wilk test. Data with normal and non-normal distributions and categories were expressed as mean ± standard deviation (SD), median with 25 and 75 percentiles, and percentage, respectively. We employed Spearman’s correlation coefficient because many variables exhibited non-normal distributions. To account for the differing degrees of reliability among the dependent variables, such as blastocyst formation rate, developmental stage, quality grade, implantation success rate, and live birth rate, weighted Spearman’s rank correlation coefficients were calculated. The weights were defined as follows: the number of oocytes used in conventional IVF (cIVF) for blastocyst formation rate, developmental stage, and quality grade, and the number of embryo transfer attempts for implantation success and live birth rates. Subsequently, a bootstrap approach with 2500 resamples was used to estimate the 95% confidence intervals. Statistical significance was set at *p*-values of <0.05.

## 3. Results

### 3.1. Background Characteristics of the Study Participants

Participant flow is shown in Supplemental Figure S1. Of the 41 enrolled couples, 21 underwent cIVF. One did not reach embryo transfer due to blastocyst development failure, yielding 20 transfers. Two were lost to follow-up, resulting in 18 couples available for live birth analysis. As shown in Table 1, 67% of the participants and 71% of their partners were ≤40 years of age. Most participants had no comorbidities. Table 2 summarizes the participants’ metabolic parameters. Most of them were not obese, and SAF values were within the age-adjusted normal range. Serum d-ROMs levels were normal in 48% of the participants, slightly high in 24%, and high in 28%. Serum glucose levels were normal in all participants, while serum low-density lipoprotein cholesterol (LDL-C), high-density lipoprotein cholesterol (HDL-C), triglycerides (TG), Zn, and free testosterone were normal in 81%, 76%, 62%, 48%, and 76% of the participants, respectively. Lifestyle habits are presented in Table 3. Cronbach’s α was calculated for the responses to this questionnaire, which was 0.77, indicating acceptable internal consistency. Approximately 50% of the participants exercised regularly, 65% were non-smokers, and 48% did not drink regularly. More than half reported not experiencing mental stress and having healthy dietary habits.

### 3.2. Background Sperm Parameters and cIVF Outcomes

Sperm parameters are presented in Table 4. Total sperm count was normal in all participants according to WHO criteria [12]. Total motility and progressive motility of sperm were within the normal ranges in 90% of cases. Sperm DFI was classified as mild in 76%, moderate in 19%, and severe in 5% of the participants. ORP levels were within the normal range in all participants. Blastocyst formation was observed in 32% of the oocytes used for cIVF. Among the formed blastocysts, good blastocyst development stage (stage ≥ 4) and quality grade (score = 3) were observed in 71% and 39%, respectively. After embryo transfer, implantation success and live birth rates were 53% and 43%, respectively.

### 3.3. Correlation of Metabolic, Lifestyle, and Sperm Factors with cIVF Outcomes

Next, correlation of metabolic, lifestyle, and sperm factors with cIVF outcomes was assessed. Female factors, including age and AMH (1.94 ng/mL [0.75–4.22]), were also included in this analysis. As shown in Table 5, sperm DFI was negatively correlated with blastocyst formation rate, development stage, and quality grade. In addition, male age was also negatively associated with blastocyte quality grade. In contrast, none of the parameters were associated with implantation success or live birth success rates.

### 3.4. Correlation of Metabolic, Lifestyle, and Sperm Factors with Sperm DFI

As shown in Supplemental Table S1, among metabolic, lifestyle, and sperm factors, serum HDL-C level was the sole male factor associated with sperm DFI (*ρ* = 0.52, *p* = 0.02).

## 4. Discussion

ART is a widely utilized therapeutic strategy for assisting infertility in many countries [2,3,4]. While the acquisition of well-developed embryos through IVF is one of the key determinants of ART success [6], the contribution of specific male factors remains incompletely understood. This study aimed to identify male factors associated with embryo formation and quality in cIVF. In the present study, most participants exhibited generally normal metabolic parameters, except for high ratio of hypertriglyceridemia, and maintained healthy lifestyle habits. Regarding sperm parameters, total sperm count was normal in all participants, and other sperm parameters, including DFI, were mostly within normal ranges. In this population, regression analyses revealed that, among evaluated metabolic, lifestyle, and sperm parameters, sperm DFI was the only factor that correlated with all blastocyte outcomes, including impaired embryo formation and quality in cIVF. Recent studies have reported that, in men with infertility, sperm DNA fragmentation index (DFI) is associated with fertilization ability independently of conventional sperm parameters, such as concentration, motility, and morphology [18,19]. Furthermore, in men diagnosed with male infertility, high sperm DFI (>30%) has been reported to be associated with lower high-quality embryo rates, blastocyst development rates, and implantation success rates in IVF compared with mild (<15%) or moderate (15–30%) DFI [15,16]. However, it remains unclear whether sperm DFI is also associated with IVF outcomes in men with non-severe DFI, particularly those with relatively preserved spermatogenesis. The present findings suggest that, although unmeasured confounding or reverse causality cannot be excluded, sperm DFI may be associated with blastocyst formation and quality in cIVF, even among normozoospermic men with non-severe DFI.

Several studies have reported male factors associated with sperm DFI, such as age, BMI, and smoking habits [15,20]; however, conflicting results have been reported [21,22]. In the present study, only serum HDL-C levels were associated with sperm DFI, consistent with the findings of our original study [9]. HDL is a lipoprotein that transports cholesterol from peripheral tissues to the liver [23]. In a previous study evaluating the association between serum lipids and sperm parameters, abnormal sperm morphology was more frequent in men with HDL-C levels > 57 mg/dL or <42 mg/dL [24]. Another study assessing both blood and seminal lipids has reported that seminal plasma contains HDL-C at approximately 25% of serum levels and that seminal HDL-C levels are higher in men with sperm abnormalities than in those without them [25]. These previous findings support the observed association between serum HDL-C and sperm DFI in this study. However, the molecular mechanisms linking serum HDL-C and sperm DFI remain unclear.

Sperm is known to be vulnerable to oxidative stress, exposure to which can cause DNA fragmentation [26,27]. In this analysis, systemic and seminal oxidative stress were assessed as serum d-ROMs and ORP, respectively. Additionally, skin levels of AGEs, which form and accumulate with aging and lifestyle factors and generate oxidative stress through interactions with their cell-surface receptor, were measured [28,29,30]. Several types of AGEs exhibit autofluorescence, and their accumulation in the skin can be quantified non-invasively as SAF. Indeed, numerous studies have reported associations between SAF levels and various non-communicable diseases [30]. However, none of these parameters were associated with sperm DFI. Consistently, a previous study also reported no correlation between systemic oxidative stress levels and sperm DFI [31]. In our previous animal study using a mouse model of diabetes and obesity, glyceraldehyde-derived AGEs, one of the most toxic AGE subtypes, accumulated in the interstitium of the testis, accompanied by increased oxidative stress levels, but these changes were not observed in the seminiferous tubules [32]. The seminiferous tubules are protected from circulating factors by the blood–testis barrier; therefore, systemic oxidative stress and AGEs may not be directly associated with sperm DFI. Regarding seminal oxidative stress, ORP levels have been reported to be positively associated with sperm DFI in men with infertility [33]. However, in the present study, sperm parameters were normal in most participants, and ORP levels were not elevated in any participants. This may account for the absence of an association between sperm DFI and ORP.

Many clinical studies have investigated whether sperm DFI is associated with implantation and pregnancy success after embryo transfer; however, findings have been conflicting. A recent meta-analysis of 92 studies reported a negative association between sperm DFI and pregnancy success in 35 studies, whereas no association was found in the remaining 57 studies [34]. In the present study, no male factors, including sperm DFI, were identified as being associated with pregnancy success or live birth after embryo transfer. In cIVF, blastocysts were formed in 32% of the oocytes, and good blastocyst development and quality were achieved in 71% and 39% of formed blastocysts, respectively. This allowed the selection of high-quality blastocysts with a greater likelihood of implantation success and live birth, which may explain why sperm DFI was associated with blastocyst formation and quality but not directly with implantation success after embryo transfer.

Previous studies have demonstrated that female reproductive factors, including ovarian reserve, oocyte quality, and underlying gynecologic conditions, can influence embryo formation and quality [6]. Although these factors are clinically important, the present subanalysis was not designed to specifically evaluate the contribution of female factors to ART outcomes. However, we examined key female parameters to provide a balanced perspective. In this cohort, female age and AMH did not show clear associations with blastocyst development, implantation, or live birth. One possible explanation is that most female partners were younger than 40 years and exhibited a relatively narrow distribution of AMH levels, which limited the variability needed to detect meaningful statistical associations. Although several participants had gynecological conditions such as endometriosis or uterine fibroids, the number of such cases was too small to assess their potential influence. Taken together, the restricted variability in female parameters, the small sample size, and the inability to conduct multivariable analyses likely reduced the statistical power to detect weak-to-moderate associations. These considerations suggest that the absence of strong correlations should not be interpreted as evidence that female factors are unimportant but rather as a limitation of this exploratory analysis, whose primary aim was to examine the association between male parameters and ART outcomes.

As a limitation of this study, IVF was performed with the conventional method [5]. Therefore, it remains unclear whether a similar association would be observed with other IVF methods, such as intracytoplasmic sperm injection. Second, sperm DFI was assessed using sperm smears with the TUNEL method. Other methods for evaluating sperm DNA fragmentation, such as the sperm chromatin structure assay and the sperm chromatin dispersion assay, are widely used in clinical settings due to their simpler procedures [34]. However, these assays must be performed immediately after sperm collection, whereas the TUNEL assays can be applied to sperm smear samples, allowing retrospective evaluation of sperm quality [35]. Nevertheless, it should be noted that the TUNEL assays are reported to have relatively lower sensitivity and may not fully capture the spectrum of DNA damage in spermatozoa. Therefore, the associations observed in this subanalysis may not necessarily be replicated when sperm DFI is assessed using other methodologies. Third, we evaluated Zn levels in serum samples. Zinc is an essential trace element, and its concentration in seminal plasma has been reported to be associated with sperm function. Several studies have demonstrated that Zn supplementation improves sperm function in individuals with infertility [36], thus suggesting a possible relationship between Zn levels in blood and those in semen. In our cohort, approximately half of the participants showed serum Zn levels below the normal range. However, it remains unclear how these serum Zn levels relate to Zn concentrations in semen, and correlation between serum and seminal zinc levels would have provided a more comprehensive understanding of the role of zinc in male infertility. Fourth, we evaluated testicular functions using serum-free testosterone levels. Measuring free testosterone has certain advantages over total testosterone because it is less affected by sex hormone-binding globulin changes that occur in obesity, diabetes, and liver dysfunction [37]. However, total testosterone remains the most widely validated marker for assessing testicular function [37]. Moreover, in the present study, free testosterone was measured using a radioimmunoassay due to local availability, a method that is not commonly used in many regions. Taken together, our data suggests that late-onset hypogonadism is unlikely to account for the observed findings, although these methodological considerations should be kept in mind when interpreting the results. Fifth, sperm morphology was not evaluated in the present study. Therefore, we were unable to determine whether sperm morphology is associated with sperm DFI or with IVF outcomes. Sixth, we found that sperm DFI was negatively correlated with blastocyst outcomes after cIVF. However, as this study was designed as an exploratory subanalysis, no single primary hypothesis was prespecified. Therefore, the possibility of confounding or reverse causality cannot be excluded, and the findings should be interpreted as hypothesis-generating. Seventh, this study had limited statistical power due to its small sample size and did not include adjustment for multiple testing to reduce the risk of type I errors, nor multivariable analyses to account for potential confounding male and female factors. In addition, methodological literature indicates that stable correlation estimates typically require sample sizes of at least 150 [38]. Therefore, the estimates observed in this study, which are based on only 21 participants, are likely unstable with wide confidence intervals, further limiting their interpretability. These limitations indicate that the present findings should be interpreted with caution and confirmed in larger, adequately powered studies to provide more robust evidence.

## 5. Conclusions

The present subanalysis suggests the hypothesis that sperm DNA fragmentation may be negatively associated with the formation of high-quality embryos in cIVF, even among normozoospermic men with non-severe levels of sperm DFI. Sperm DFI may represent a potential research marker for embryo development in cIVF, although its clinical relevance remains uncertain and requires confirmation in larger, adequately powered studies.

## Figures and Tables

**Table 1:** Background characteristics of the study participants.

**Participants (Men)**		**Partners (Women)**	
Number	21	Number	21
Age (years old)	38.9 ± 6.0	Age (years old)	36.3 ± 5.1
Comorbidities		Comorbidities	
Hyperuricemia (*n*)	1 [4.8%]	Ovulation dysfunction (*n*)	4 [19.1%]
Hypercholesterolemia (*n*)	1 [4.8%]	Uterine fibroids (*n*)	4 [19.1%]
Hypertension (*n*)	1 [4.8%]	Endometriosis (*n*)	3 [14.3%]
		Polycystic ovary syndrome (*n*)	1 [4.8%]
		Adenomyosis (*n*)	1 [4.8%]
		Sexual dysfunction (*n*)	1 [4.8%]

Square brackets indicate percentages. *n* represents the number of subjects.

**Table 2:** Clinical parameters of the study participants.

Height (cm)	171.5 ± 5.7
Body weight (kg)	69.0 ± 11.5
BMI (kg/m^2^)	23.4 ± 3.2
Wasit circumstance (cm)	83 [78–91]
SAF (AU)	1.7 [1.6–1.9]
Serum d-ROMs (U.CARR)	309 ± 55
Serum glucose (mg/dL)	103 [99–117]
Serum LDL-C (mg/dL)	113 ± 30
Serum HDL-C (mg/dL)	53 ± 16
Serum TG (mg/dL)	168 ± 89
Serum Zn (ug/dL)	77 [69–89]
Serum-free testosterone (pg/mL)	11.8 ± 3.4

Data with normal and non-normal distributions and categorical data were expressed as mean ± standard deviation (SD), median with 25th and 75th percentiles, and percentage, respectively. BMI: body mass index, SAF: skin autofluorescence intensity, d-ROMs: diacron-reactive oxygen metabolites, LDL-C: low-density lipoprotein cholesterol, HDL-C: high-density lipoprotein cholesterol, TG: triglycerides, Zn: zinc.

**Table 3:** Lifestyle habits of the study participants.

A. How often do you engage in physical activity, such as a 30 min walk or equivalent exercise				
1. Not at all	2. No exercise, but walk around at home or office	3. Once a week	4. 2–3 times a week	5. More than 4 times a week
3 [14.3%]	8 [38.1%]	3 [14.3%]	4 [19.0%]	3 [14.3%]
B. How long have you been smoking?				
1. Daily smoking for ≥10 years	2. Daily smoking for <10 years	3. Quit smoking within 1 year	4. Quit smoking more than 1 year ago	5. Never smoked
6 [28.6%]	1 [4.8%]	2 [9.5%]	1 [4.8%]	11 [52.4%]
C. How frequently do you consume alcoholic beverages?				
1. More than 4 times a week	2. 2–3 times a week	3. Once a week	4. Sometimes	5. Never
6 [28.6%]	2 [9.5%]	3 [14.3%]	6 [28.6%]	4 [19.0%]
D. How many hours do you sleep each day?				
1. Less than 4 h	2. 4–5 h	3. 5–7 h	4. 7–8 h	5. More than 8 h
3 [14.3%]	8 [38.1%]	0 [0%]	9 [42.9%]	1 [4.8%]
E. Do you feel mentally stressed?				
1. Strongly agree	2. Agree	3. Neutral	4. Disagree	5. Strongly disagree
1 [4.8%]	7 [33.3%]	0 [0%]	11 [52.4%]	2 [9.5%]
F. Do you eat plenty of vegetables?				
1. Strongly disagree	2. Disagree	3. Neutral	4. Agree	5. Strongly agree
2 [9.5%]	3 [14.3%]	0 [0%]	6 [28.6%]	10 [47.6%]
G. Do you eat breakfast daily?				
1. Strongly disagree	2. Disagree	3. Neutral	4. Agree	5. Strongly agree
6 [28.6%]	3 [14.3%]	1 [4.8%]	4 [19.0%]	7 [33.3%]
H. Do you try to avoid overeating beyond about 80% fullness?				
1. Strongly disagree	2. Disagree	3. Neutral	4. Agree	5. Strongly agree
3 [14.3%]	4 [19.0%]	2 [9.5%]	10 [47.6%]	2 [9.5%]
I. Do you try to avoid consuming greasy foods?				
1. Strongly disagree	2. Disagree	3. Neutral	4. Agree	5. Strongly agree
6 [28.6%]	6 [28.6%]	6 [28.6%]	2 [9.5%]	1 [4.8%]
J. Do you try to avoid consuming processed foods?				
1. Strongly disagree	2. Disagree	3. Neutral	4. Agree	5. Strongly agree
5 [23.8%]	5 [23.8%]	4 [19.0%]	5 [23.8%]	2 [9.5%]
K. Do you try to avoid consuming sugary foods, such as cakes and candies?				
1. Strongly disagree	2. Disagree	3. Neutral	4. Agree	5. Strongly agree
6 [28.6%]	7 [33.3%]	0 [0%]	2 [9.5%]	6 [28.6%]
L. Do you eat vegetables first during your meals?				
1. Strongly disagree	2. Disagree	3. Neutral	4. Agree	5. Strongly agree
2 [9.5%]	3 [14.3%]	4 [19.0%]	7 [33.3%]	5 [23.8%]

Values show the number of participants with the percentage in square brackets.

**Table 4:** Background sperm parameters and in vitro fertilization outcomes.

Semen volume (mL)	3.3 ± 1.1
Sperm concentration (10^6^/mL)	130 [101–201]
Total sperm count (×10^6^ per ejaculate)	432 [261–666]
Sperm total motility (%)	64 ± 15
Sperm progressive motility (%)	55 ± 16
ORP (mV/10^6^ sperm/mL)	0.22 [0.17–0.41]
Sperm DFI (%)	6.5 [3.0–15.4]
Total number of retrieved oocytes	14.8 ± 10.0
Total number of mature oocytes	4.3 ± 3.2
Blastocyst formation rate (%)	32 ± 23
Blastocyst development stage	2.6 ± 1.4
Blastocyst quality grade	1.3 ± 0.9
Total number of embryo transfers	2.6 ± 1.9
Implantation success after embryo transfer (%)	53 ± 42
Live birth after embryo transfer (%)	43 ± 44
Miscarriage after implantation success (%)	22 ± 39

Data with normal and non-normal distributions were expressed as mean ± SD and median with 25th and 75th percentiles, respectively. Data on blastocyst growth stage, blastocyst quality grade, and pregnancy success after embryo transfer were available for 18 participants due to blastocyst formation failure in 3 cases. ORP, oxidation–reduction potential; DFI, DNA fragmentation index.

**Table 5:** Correlation of metabolic, lifestyle, and sperm factors associated with blastocyst outcomes.

	**Blastocyst Formation Rate**		**Blastocyst Development Stage**		**Blastocyst Quality Grade**	
**Variables**	***ρ***	***p***	***ρ***	***p***	***ρ***	***p***
Male age (years old)	−0.01 [−0.37–0.37]	0.98	−0.38 [−0.63–−0.07]	0.09	−0.50 [−0.74–−0.18]	0.02
Female age (years old)	−0.20 [−0.54–0.16]	0.38	−0.27 [−0.59–0.11]	0.23	−0.31 [−0.60–0.05]	0.18
Female AMH (ng/mL)	−0.01 [−0.36–0.42]	0.97	0.13 [−0.25–0.51]	0.57	0.10 [−0.30–0.50]	0.68
BMI (kg/m^2^)	0.06 [−0.38–0.49]	0.78	0.17 [−0.26–0.58]	0.45	0.24 [−0.17–0.59]	0.30
Wasit circumstance (cm)	−0.03 [−0.45–0.42]	0.90	−0.06 [−0.44–0.37]	0.81	−0.03 [−0.40–0.35]	0.90
SAF (AU)	−0.07 [−0.47–0.36]	0.77	−0.29 [−0.66–0.11]	0.20	−0.39 [−0.70–0.03]	0.08
Serum d-ROMs (U.CARR)	0.06 [−0.34–0.48]	0.81	−0.03 [−0.41–0.38]	0.90	−0.09 [−0.42–0.30]	0.71
Serum glucose (mg/dL)	0.06 [−0.36–0.45]	0.81	0.11 [−0.27–0.49]	0.63	0.16 [−0.27–0.54]	0.50
Serum LDL-C (mg/dL)	−0.10 [−0.49–0.31]	0.67	0.04 [−0.39–0.46]	0.86	−0.01 [−0.43–0.40]	0.95
Serum HDL-C (mg/dL)	−0.27 [−0.62–0.13]	0.24	−0.39 [−0.75–0.06]	0.08	−0.39 [−0.75–0.09]	0.08
Serum TG (mg/dL)	0.21 [−0.25–0.61]	0.36	0.04 [−0.40–0.48]	0.87	0.12 [−0.31–0.53]	0.59
Serum Zn (ug/dL)	0.14 [−0.22–0.49]	0.52	0.17 [−0.16–0.56]	0.30	0.24 [−0.56–0.56]	0.31
Serum-free testosterone (pg/mL)	−0.08 [−0.54–0.35]	0.71	−0.03 [−0.47–0.35]	0.87	−0.06 [−0.43–0.36]	0.83
Exercise-related lifestyle habits	0.36 [0.01–0.63]	0.11	0.11 [−0.24–0.46]	0.62	0.19 [−0.15–0.48]	0.40
Smoking-related lifestyle habits	−0.33 [−0.66–0.10]	0.15	−0.38 [−0.69–0.01]	0.09	−0.29 [−0.63–0.10]	0.20
Alcohol-related lifestyle habits	−0.10 [−0.49–0.33]	0.67	0.12 [−0.27–0.48]	0.61	0.04 [−0.33–0.41]	0.87
Mental stress-related lifestyle habits	−0.05 [−0.37–0.27]	0.82	0.06 [−0.29–0.40]	0.80	0.13 [−0.25–0.50]	0.59
Diet-related lifestyle habits	0.17 [−0.30–0.63]	0.47	0.04 [−0.39–0.43]	0.87	0.07 [−0.34–0.46]	0.76
Sperm concentration (10^6^/mL)	0.23 [−0.18–0.60]	0.31	0.22 [−0.22–0.60]	0.33	0.10 [−0.32–0.49]	0.66
Total sperm count (×10^6^ per ejaculate)	0.13 [−0.27–0.48]	0.57	0.13 [−0.30–0.55]	0.56	0.00 [−0.40–0.40]	0.99
Sperm total motility (%)	−0.03 [−0.42–0.36]	0.90	0.28 [−0.07–0.60]	0.22	0.14 [−0.25–0.49]	0.53
Sperm progressive motility (%)	0.00 [−0.41–0.39]	1.00	0.34 [−0.04–0.65]	0.14	0.16 [−0.24–0.51]	0.48
Sperm ORP (mV/106 sperm/mL)	−0.15 [−0.49–0.22]	0.51	−0.11 [−0.49–0.31]	0.63	−0.05 [−0.47–0.39]	0.82
Sperm DFI (%)	−0.50 [−0.78–−0.11]	0.02	−0.45 [−0.71–−0.15]	0.04	−0.45 [−0.70–−0.08]	0.04

All data presented refer to male parameters, except for female age. *ρ* values represent the point estimates of Spearman’s correlation coefficients with their 95% confidence intervals.

## Data Availability

The datasets generated and/or analyzed during the present study are not publicly available because data sharing was not included in the consent form. However, they are available from the corresponding author upon reasonable request. The raw data are securely maintained in a controlled-access database system managed by Showa Medical University School of Medicine.

## Supplementary Materials

The following supporting information can be downloaded at: https://www.mdpi.com/article/10.3390/jcm14248892/s1, Supplemental Figure S1. Study flow chart. Supplemental Table S1. Correlation of metabolic, lifestyle, and sperm factors associated with sperm DFI.
