# Supplementary material for: Sperm DNA Fragmentation in Normozoospermic Men Is Associated with Blastocyst Formation and Quality in Conventional In Vitro Fertilization
Source: J Clin Med. 2025 Dec 16;14(24):8892. doi: 10.3390/jcm14248892 (PMC12734054; doi:10.3390/jcm14248892)
Supplement: Supplementary file 1 [file jcm-14-08892-s001.zip › jcm-4009507-supplementary.pdf]

Journal of
Clinical Medicine

Supplemental Figure S1. Study flow chart.

Flowchart illustrating participant eligibility and inclusion into the cIVF cohort. Of the 41 eligible couples, 21 underwent conventional IVF. One couple did not proceed with embryo transfer due to blastocyst development failure, resulting in 20 embryo transfers. Two couples were lost to follow-up before pregnancy outcome was confirmed; therefore, 18 couples were included in the live birth analysis.

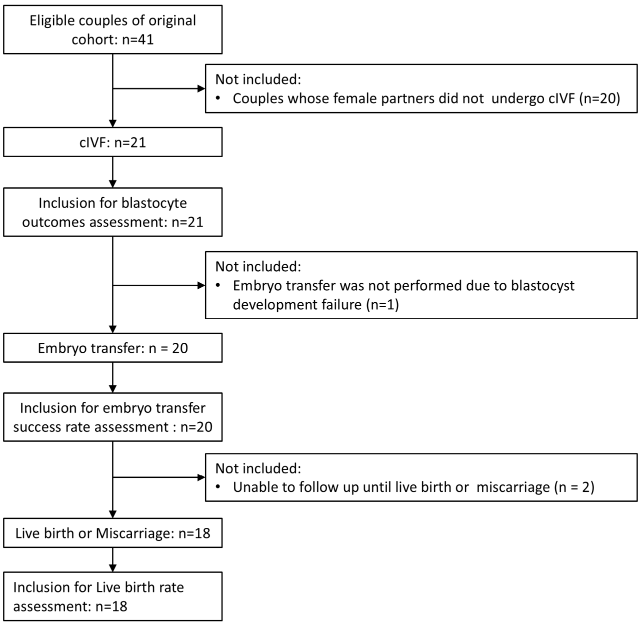

Supplemental Table S1. Correlation of metabolic, lifestyle, and sperm factors associated with sperm DFI.

Variables	ρ	p
Male age (years old)	0.33 [-0.11 – 0.66]	0.15
BMI (kg/m2)	-0.16 [-0.54 – 0.23]	0.47
Wasit circumstance (cm)	-0.30 [-0.65 – 0.10]	0.19
SAF (AU)	0.38 [-0.05 – 0.75]	0.09
Serum d-ROMs (U.CARR)	0.33 [-0.09 – 0.69]	0.14
Serum glucose (mg/dL)	-0.12 [-0.50 – 0.27]	0.59
Serum LDL-C (mg/dL)	-0.22 [-0.55 – 0.15]	0.35
Serum HDL-C (mg/dL)	0.52 [0.18 – 0.75]	0.02
Serum TG (mg/dL)	-0.35 [-0.69 – 0.04]	0.12

Serum Zn (ug/dL)	-0.06 [-0.46 – 0.32]	0.77
Serum free testosterone (pg/mL)	-0.04 [-0.41 – 0.39]	0.88
Exercise-related lifestyle habits	-0.33 [-0.65 – 0.07]	0.15
Smoking-related lifestyle habits	0.21 [-0.17 – 0.56]	0.35
Alcohol-related lifestyle habits	-0.17 [-0.54 – 0.25]	0.46
Mental stress–related lifestyle habits	-0.10 [-0.46 – 0.26]	0.66
Diet-related lifestyle habits	-0.01 [-0.41 – 0.40]	0.96
Sperm concentration (10^6/mL)	-0.24 [-0.63 – 0.16]	0.29
Total sperm count (×10^6 per ejaculate)	-0.39 [-0.73 – 0.00]	0.08
Sperm total motility (%)	-0.38 [-0.79 – 0.05]	0.09
Sperm progressive motility (%)	-0.29 [-0.71 – 0.15]	0.20
Sperm ORP (mV/10^6 sperm/mL)	0.27 [-0.13 – 0.65]	0.24

ρ values represent the point estimates of Spearman's correlation coefficients with their 95% confidence intervals.
